# Supplementary material for: The Expression of TP63 as a Biomarker of Early Recurrence in Resected Esophageal Squamous Cell Carcinoma after Neoadjuvant Chemoradiotherapy
Source: Biomedicines. 2024 May 16;12(5):1101. doi: 10.3390/biomedicines12051101 (PMC11117789; doi:10.3390/biomedicines12051101)
Supplement: Supplementary file 1 [file biomedicines-12-01101-s001.zip › biomedicines-2994836-supplementary.pdf]

Table S1. Characteristics of the enrolled 50 ESCC patients form IHC.

Characteristics	Total	Recurrence	Non-Recurrence	*p*
**No. of patients**	N = 50	N = 25	N = 25	
**Age (year, mean)**	54.52 ± 7.71	53.32 ± 7.81	55.72 ± 7.57	0.28 [a]
**Gender**				
**Male**	48 (98.0%)	23 (92.0%)	25 (100.0%)	
**Female**	2 (4.0%)	2 (8.0%)	0 (0.0%)	
**Pathologic stage** [c]				
**I**	1 (2.0%)	0 (0.0%)	1 (100%)	0.237 [b]
**IA**	7(14.0%)	3 (42.9%)	4 (57.1%)	
**IA2**	1 (2.0%)	1 (100%)	0 (0.0%)	
**II**	1 (2.0%)	1 (100%)	0 (0.0%)	
**IIA**	3 (6.0%)	1 (33.3%)	2 (66.7%)	
**IIB**	16 (32.0%)	4 (25.0%)	12 (75.0%)	
**IIIA**	8 (16.0%)	6 (75.0%)	2 (25.0%)	
**IIIB**	7 (14.0%)	5 (71.4%)	2 (28.6%)	
**IIIC**	2 (4.0%)	2 (100%)	0 (0.0%)	
**IVA**	2 (4.0%)	1 (50.0%)	1 (50.0%)	

[a] *t*-test analyzed the differences of age between patients with recurrence and non-recurrence ECSS patients.
[b] Chi-square test analyzed the differences in each clinical stage between two groups.
[c] Total *n* = 48 due to missing data for 2 patients.
